# The Scientific Response to Zika Virus

**DOI:** 10.3390/jcm8030369

**Published:** 2019-03-15

**Authors:** Miguel A. Martín-Acebes, Juan-Carlos Saiz

**Affiliations:** Department of Biotechnology, Instituto Nacional de Investigación y Tecnología Agraria y Alimentaria (INIA), 28040 Madrid, Spain; jcsaiz@inia.es

**Keywords:** Zika virus, epidemics, scientific literature, antiviral, vaccines

## Abstract

Zika virus (ZIKV) is a mosquito-borne pathogen that became renowned in 2015 in Brazil mainly due to its association with microcephaly in newborns. Although most infections in adults are asymptomatic or cause mild illnesses, in a reduced number of cases, ZIKV can also produce severe complications that include neurological disorders (Guillain–Barré syndrome), ocular lesions, or reproductive alterations. From 2015 the efforts of a significant part of the scientific community were placed on ZIKV research, which has resulted in an unpredicted escalation of the knowledge of the biology and pathology of this virus. The rapid response of the scientific community against ZIKV highlights its enormous potential to counter attack a viral threat within a short time period. It is expected that this huge collaborative effort will produce affordable and effective prophylactic and therapeutic tools against ZIKV. Nevertheless, there are still other arboviral threats different from ZIKV that should not be forgotten.

Zika virus (ZIKV) is a flavivirus transmitted by mosquitoes that jumped to fame in 2015 mainly due to its association with neonatal malformations (i.e., microcephaly) in Brazil [1]. Although ZIKV is responsible for congenital Zika syndrome, most infections are asymptomatic or only cause mild illnesses characterized by rush, conjunctivitis, muscle and joint pain, malaise, or headache. In a reduced number of cases, ZIKV can also produce severe complications that include neurological disorders (Guillain–Barré syndrome) and ocular lesions [2,3]. The virus can also be sexually transmitted and can persist in the male genital tract, which could lead to infertility [4]. After its explosion in Brazil, the virus rapidly spread through Latin America, leading to the declaration of a Public Health Emergency of International Concern (PHEIC) by the World Health Organization (WHO) in February 2016. During the first stages of the epidemic, titanic efforts of the scientific community were conducted to rapidly understand ZIKV biology and pathology, improve diagnostic methodologies, and develop specific therapeutic and prophylactic alternatives. In fact, bibliometric analyses of the impact of ZIKV on scientific literature showed a huge increment in ZIKV-related scientific literature and patent application after 2015 [5,6,7,8]. For the non-specialist, this can be easily and rapidly visualized by searching for Zika virus or ZIKV at PubMed, which is the most commonly used engine to access the MEDLINE database of references and abstracts on life sciences and biomedical topics (Figure 1A). However, what seems to be more surprising is that when similar searches were performed for other medically relevant arthropod-borne viruses (arboviruses) namely, Dengue virus (DENV), Yellow fever virus (YFV), West Nile virus (WNV), and Chikungunya virus (CHIKV), it was observed that the document count for ZIKV over the last three years extensively surpassed them. Remarkably, this is even the case for DENV, which is currently considered the most life-threatening arbovirus, accounting for up to 100 million infections each year and a leading cause of serious illness and death among children [9]. Thus, it could seem that the response against ZIKV has been overdimensioned in comparison to other viral threats. This may be the product of a wide variety of factors, including the social alarm caused by babies with microcephaly, the rapid spread of the virus, the declaration of the PHEIC, the facilitation of ZIKV-research by funding agencies, or the interest of researchers to walk into an unexplored field. What it is clear is that the interest of the research community on ZIKV grew to unexpected levels after the 2015 epidemic. This can be again easily exemplified by the comparison of the research results for ZIKV after the Brazilian outbreak in 2015 with other recent viral outbreaks such as that of WNV in 1999 in North America [10], CHIKV in 2004–2005 southwestern Indian Ocean region, India, and Southeast Asia [11], or Ebola virus (EBOV) in 2014 in Western Africa [12].

Has this increase in ZIKV-interest been translated into practical solutions? In our opinion, the answer is yes. For instances, the catalogue of options for molecular diagnostic and reliable serological tests has been greatly expanded after the ZIKV uprising in Brazil [13]. Moreover, important milestones of ZIKV research were rapidly achieved. One of them was the development of amenable animal models. Although non-human primates are naturally susceptible to ZIKV infection, ethical, financial, and operative concerns limited their utilization in ZIKV-research. The utilization of immuno-deficient mice solved the problems with the “resistance” to peripheral ZIKV infection in adult mice and provided useful animal models for ZIKV infection [14]. These mice models have contributed to the advancement of the understanding of the pathology of ZIKV, antiviral testing, and vaccine evaluation. The utilization of 3D culture systems (brain organoids) also contributed to the understanding of ZIKV pathology [15]. On combining clinical findings, animal, organoid, and cell culture results, ZIKV-induced microcephaly was found to be the result of the marked neurotropism of ZIKV. This pathogen is able to cross the placental barrier and infect neural progenitor cells and neurons, causing premature differentiation, inducing apoptosis, and thus reducing brain size. In addition to this “direct effect”, placental insufficiency and inflammatory responses during the infection would also contribute to intrauterine growth restriction [16]. However, it should be highlighted that critical questions about ZIKV biology and its pathogenesis are not yet fully understood and although there are working hypotheses to explain the development of microcephaly, the complete puzzle has not been totally solved. Regarding therapeutic approaches, a wide panel of substances with antiviral activity against this virus was rapidly identified [17] and is still growing day by day. One of the most promising approaches for the rapid identification of drugs effective against ZIKV was carried out by drug repositioning studies screening libraries of drugs approved for human use, a methodology that brings the advantage of the identification of compounds with documented security. Additionally, the search for novel and specific drugs has already identified a wide variety of cellular and viral targets suitable for pharmacological intervention. The structure of some of the ZIKV proteins has been solved, which probably accelerates both drug discovery and vaccine design [18]. This search for antivirals is also contributing to the identification of broad-spectrum antiviral candidates that could be effective against other medically relevant flaviviruses like DENV or WNV, and could even help to combat other still undefined future arboviral threats [19]. Notwithstanding, we have to be still cautious when thinking in antiviral prescription against ZIKV, because there is yet no specific antiviral drug licensed to combat any flavivirus. Moreover, antiviral therapies against ZIKV will have to face extra challenges such as safety in pregnant women or its ability to cross the blood–brain barrier to inhibit virus infection in nervous tissues [20]. In this scenario, vaccination appears to be the most feasible control strategy in the short term. In fact, there are already multiple vaccine candidates that have undergone phase I clinical trials, and even one DNA vaccine that has entered into Phase II trials [21]. The good results obtained with vaccination for the control of other flaviviral diseases like yellow fever, Japanese encephalitis, or tick-borne-encephalitis invite optimism. However, the experience with DENV vaccination indicated that vaccine performance may depends on serostatus, showing that vaccination against certain flaviviruses is not a bed of roses [22]. Results from animal experimentation suggest that the immunological crosstalk between ZIKV and other flaviviruses (i.e., DENV) could complicate massive utilization of some of these vaccines [23,24]. In fact, there are experimental evidences of ZIKV-vaccine candidates that induce a generation of cross-reactive antibodies that may enhance DENV infection, although there are promising approaches to minimize this possibility [25]. Additionally, there is anecdotal evidence showing that neutralizing antibodies if induced against ZIKV and DENV at high titers could potentially prevent enhancement of infection [26,27]. Thus, in order to develop safe ZIKV vaccines, an important effort is urgently required to assess the real potential of antibody-dependent enhancement of infection within the populations suitable for ZIKV-vaccination [28].

In summary, the response against ZIKV highlights the enormous potential of the scientific community to counter-attack against a viral threat. It is expected that this huge collaborative effort will produce the desired results and that we will have affordable and effective prophylactic and therapeutic tools against ZIKV in a reasonable period of time. Nevertheless, we would like it to be remembered that there are still arboviral threats other than ZIKV that should not be forgotten.

## Figures and Tables

**Figure 1:**
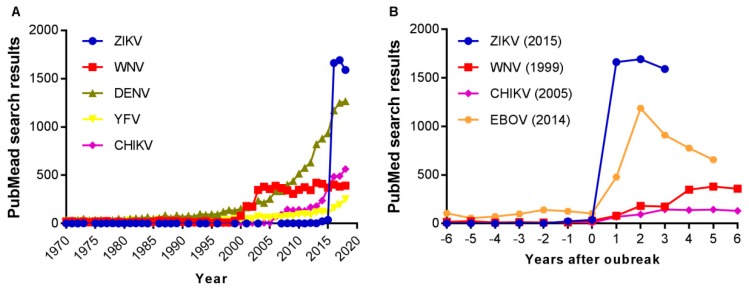
Impact of Zika virus (ZIKV) on scientific literature. (**A**) The graph displays the number of search results for Zika virus or ZIKV retrieved from PubMed using the “Results by year” tool. For comparison, similar searches were performed for the other flaviviruses displayed. Data corresponds to searches performed on 30 January 2019. (**B**) Increase in research interests after different outbreaks or epidemics of selected human pathogens. The number of search results 6 years before each outbreak and 3–6 years after is compared. The outbreaks of ZIKV in 2015 in South America, West Nile virus (WNV) in 1999 in North America, Chikungunya virus (CHIKV) in 2004–2005 in the southwestern Indian Ocean region, India, and Southeast Asia, and Ebola virus (EBOV) in 2014 in Western Africa were analyzed. Searches were performed as described in A. Data corresponds to searches performed on 30 January 2019.
